# Cotransfected human chondrocytes: over-expression of *IGF-I* and *SOX9* enhances the synthesis of cartilage matrix components collagen-II and glycosaminoglycans

**DOI:** 10.1590/1414-431X20154732

**Published:** 2015-10-06

**Authors:** M. Simental-Mendía, J. Lara-Arias, E. Álvarez-Lozano, S. Said-Fernández, A. Soto-Domínguez, G. R. Padilla-Rivas, H. G. Martínez-Rodríguez

**Affiliations:** 1 Department of Biochemistry and Molecular Medicine, Faculty of Medicine, Autonomous University of Nuevo León, Monterrey, NL, Mexico; 2 Laboratory of Tissue Engineering, Bone and Tissue Bank, Universitary Hospital, Autonomous University of Nuevo León, Monterrey, NL, Mexico; 3 Department of Histology, Faculty of Medicine, Autonomous University of Nuevo León, Monterrey, NL, Mexico

**Keywords:** Type II collagen, Glycosaminoglycans, *IGF-I*/*SOX9* transgenes, Human chondrocytes, Articular cartilage, Cotransfection

## Abstract

Damage to cartilage causes a loss of type II collagen (Col-II) and glycosaminoglycans (GAG). To restore the original cartilage architecture, cell factors that stimulate Col-II and GAG production are needed. Insulin-like growth factor I (*IGF-I*) and transcription factor *SOX9*are essential for the synthesis of cartilage matrix, chondrocyte proliferation, and phenotype maintenance. We evaluated the combined effect of *IGF-I* and *SOX9* transgene expression on Col-II and GAG production by cultured human articular chondrocytes. Transient transfection and cotransfection were performed using two mammalian expression plasmids (pCMV-SPORT6), one for each transgene. At day 9 post-transfection, the chondrocytes that were over-expressing *IGF-I*/*SOX9* showed 2-fold increased mRNA expression of the *Col-II* gene, as well as a 57% increase in Col-II protein, whereas type I collagen expression (*Col-I*) was decreased by 59.3% compared with controls. The production of GAG by these cells increased significantly compared with the controls at day 9 (3.3- *vs* 1.8-times, an increase of almost 83%). Thus, *IGF-I*/*SOX9* cotransfected chondrocytes may be useful for cell-based articular cartilage therapies.

## Introduction

Articular cartilage is an avascular and highly organized tissue characterized by a low cell density, complex biomechanical properties, and a poor capacity for healing. After injury, type II collagen (Col-II) and glycosaminoglycans (GAG), two major and essential components of the cartilage extracellular matrix, are lost. The repair tissue that forms is not hyaline cartilage, but instead a fibrocartilage rich in type I collagen (Col-I) (1), which eventually fails (2). Therefore, a method that can induce proper repair of damaged cartilage is needed. The current surgical interventions, including microfracture, chondral grafts, and chondrocyte transplantation, among others, are unable to restore the original cartilage surface (3). To re-establish the structural integrity of hyaline cartilage after injury, the transfer of genes encoding factors that increase cell proliferation and their differentiation into articular chondrocytes has been proposed (4). The regeneration of articular cartilage is a complex process that requires stimulation by several chondrogenic factors. Thus, a therapeutic strategy based on the delivery of multiple recombinant genes may induce better functional repair (5). Because of their chondro-regenerative effects, genes coding for fibroblast growth factor 2 (*FGF-2*) (6), transforming growth factor-β (*TGF-β*) (7), bone morphogenetic proteins 7 and 2 (*BMP-7* and *BMP-2*) (8), insulin-like growth factor I (*IGF-I*) (9), and the transcription factor *SOX9* (10) have been transferred to chondrocytes, both individually and in combination (5,11). Encouraging results have been reported with the use of combined, rather than single, gene transfer (4,12). However, the simultaneous effects of *IGF-I* and *SOX9*, two of the major factors involved in the matrix synthesis by chondrocytes in human articular cartilage, have not been reported. *IGF-I* induces specific anabolic effects on cartilage explants and chondrocyte monolayers. Chondrocytes transfected with the *IGF-I* gene exhibit an increased synthesis of large proteoglycan aggregates that are partially composed of GAG and Col-II (7,9). In addition, *SOX9* is a transcription factor capable of shifting the metabolic balance towards the synthesis of hyaline cartilage matrix components, as well as stimulating chondrocyte differentiation (12).

Most gene transfection for stimulating cartilage repair is done using viruses (12,13). These vectors have long-term expression, but can produce effects with undesirable consequences, such as the induction of systemic inflammatory response syndrome after systemic administration of adenoviral vectors and deregulation of T-cell proliferation driven by retrovirus enhancer activity (14). Transient cotransfection using plasmids as vectors has produced promising results (15). The plasmids remain active inside the cells for only a short, but sufficient, amount of time to express the desired genes, leading to subsequent cartilage healing (16) while reducing the potential risks to patients.

In the present study, we performed a transient cotransfection of chondrocytes with plasmids carrying the cDNA for *IGF-I* and *SOX9* to create cotransfected chondrocytes (CTC). We quantified the expression of *IGF-I* and *SOX9* and evaluated the effect of those two factors on Col-II and GAG synthesis in the CTC compared with those of non-transfected chondrocytes (NTC) and chondrocytes transfected with only one plasmid coding for *IGF-I* (*IGF-I*-TC) or *SOX9* (*SOX9*-TC). We also determined the effects of *IGF-I*, *SOX9*, or both factors on the synthesis of Col-I, which is a major component of the bone matrix, but not of hyaline cartilage (17).

## Material and Methods

### *IGF-I* and *SOX9* plasmid vectors

A pCMV-SPORT6 plasmid backbone (Open Biosystems Inc., USA) was used to construct the pCMV-SPORT6 *IGF-I* and pCMV-SPORT6 *SOX9*plasmids. *IGF-I* cDNA was cloned into the mammalian pCMV-SPORT6 plasmid using the restriction enzymes *Not*I and *Sal*I (Invitrogen, USA). The same construction process was followed to create a pCMV-SPORT6 EGFP plasmid carrying cDNA for enhanced green fluorescent protein (EGFP). The EGFP sequence was obtained from the pIRES2-EGFP plasmid (kindly provided by Dr. Martín Canizales, MD, Anderson Cancer Center, Houston, TX, USA). The vector pCMV-SPORT6 contained the selectable ampicillin-resistant gene and pUC origin, which allowed for plasmid amplification in *Escherichia coli* TOP 10. The pCMV-SPORT6 *SOX9*, pCMV-SPORT6 *IGF-I*, and pCMV-SPORT6 EGFP plasmids were purified through a silica membrane column with a Plasmid Midi Kit (Qiagen, USA).

### Human chondrocyte isolation and culture

Human chondrocytes were obtained from the unused portions of three cartilage biopsies. The cells were recovered using successive cartilage digestions with 0.25% trypsin and 2 mg of type II collagenase/mL (Sigma-Aldrich Co., USA). The chondrocytes were suspended in opti-MEM medium supplemented with 10% fetal bovine serum, gentamicin (0.05 mg/mL), and amphotericin B (50 ng/mL; complete medium), all purchased from Gibco¯ (Thermo Fisher Scientific, USA). When the monolayers reached 80% confluence, the chondrocytes were harvested, washed three times with phosphate buffered saline (Sigma-Aldrich Co.), and their concentration was adjusted to 2×10^5^ cells/mL in complete medium. Aliquots of the cell suspension were seeded into wells of a 6-well culture plate (Corning Incorporated, USA) containing 1 mL of complete medium and incubated at 37°C for 24 h in a 5% CO_2_atmosphere.

### Chondrocyte transfection

The chondrocyte monolayers at 80% confluence were transfected with a mixture of the plasmid pCMV-SPORT6 EGFP and the FuGENE 6 transfection reagent (Roche Applied Bioscience, USA) using 1:3, 2:3, and 1:6 ratios of plasmid µg to FuGENE µL and incubated at 37°C in a 5% CO_2_ atmosphere for 48 h. The number of fluorescent chondrocytes was determined using a Nikon 50i microscope with an epifluorescence illuminator (Nikon Instruments Inc., USA) at a magnification of 20×. The transfection efficiency was reported as the percentage of fluorescent cells calculated with respect to the total number of cells observed in eight randomly selected microscopic fields. This experiment was performed in triplicate.

Chondrocyte transfection with pCMV-SPORT6 *SOX9* and pCMV-SPORT6 *IGF-I* was performed using 1.0 µg of plasmid DNA and 3.0 µL of FuGENE 6 reagent. The cotransfection with both plasmids was performed using 2.0 µg of plasmid DNA (1.0 µg pCMV-SPORT6 *SOX9* and 1.0 µg pCMV-SPORT6 *IGF-I*) and 6.0 µL of FuGENE6 transfection reagent per well. These preparations were incubated for 3, 6, or 9 days at 37°C in a 5% CO_2_atmosphere. All chondrocyte transfections were performed according to the instructions provided by the FuGENE 6 transfection reagent manufacturer.

### Stimulation of proliferation by *IGF-I* and *SOX9*

We counted the numbers of NTC, *IGF-I*-TC, *SOX9*-TC, and CTC with a Nikon microscope (Nikon Instruments) at a magnification of 40× in eight randomly selected fields per microplate well.

### Total RNA extraction and RT-PCR

On days 3, 6, and 9 post-transfection (PT), the total RNA was isolated from transfected, CTC and NTC using an RNA cell and tissue purification kit (GENTRA Systems, USA). cDNA was synthesized from each RNA preparation using M-MLV reverse transcriptase (Invitrogen), and the total RNA (500 ng) was treated with 0.5 U of deoxyribonuclease I (DNase I; Invitrogen) to digest genomic DNA. The genes *SOX9*, *IGF-I*, and the constitutive gene glyceraldehyde-3-phosphate dehydrogenase (*GAPDH*) were amplified by polymerase chain reaction (PCR) using specific primers (Table 1).

**Table:**
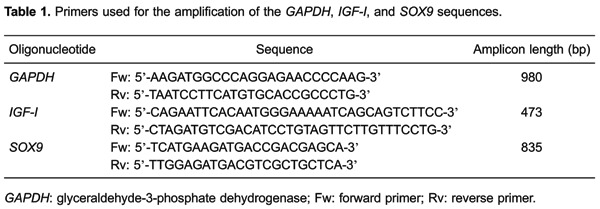


The amplification products were separated by electrophoresis in a 1.5% agarose gel (Invitrogen) and stained with 5 µL of ethidium bromide at a concentration of 0.5 mg/mL (Sigma-Aldrich Co.). The PCR products were visualized on a gel documentation system (UVP, Model M-26E; USA). A densitometric analysis was performed with the Phoretix 1D software (TotalLab Ltd., UK; available online: <http://www.totallab.com/1d-downloads/>). The density of each band was expressed as a value normalized to the average *GAPDH*cDNA band density in each gel. The density of the cDNA bands were expressed as their total number of pixels, and the level of mRNA expression was assumed to be equivalent to the density of the respective bands.

### Quantitative RT-PCR

The total RNA and cDNA were prepared according to the methods described above. The reactions were performed on a 7500 fast real-time PCR system using MicroAmp 96-well reaction plates and TaqMan Universal PCR Master Mix. Specific TaqMan probes were used to detect the expression of *Col-I*(Hs00264051_m1) and *Col-II* (Hs00264051_m1) with *GAPDH* as the internal control (Hs02758991_g1). A gene expression analysis was performed using the comparative C_T_ method (ΔΔC_T_). All instruments, laboratory materials, and chemicals used during the qRT-PCR experiments were purchased from Applied Biosystems (USA).

### Immunolabeling for Col-I and Col-II

The suspensions of CTC, *SOX9*-TC, *IGF-I*-TC, and NTC were adjusted to densities of 2×10^4^ cells/mL in complete medium, and 500 µL aliquots were seeded into each compartment of a four-well microchamber slide (NuncTM; Thermo Fisher Scientific). One microchamber slide was prepared for each transfection condition, and the microchamber slides were immediately incubated at 37°C in a 5% CO_2_ atmosphere for 3, 6, or 9 days. Next, the culture medium was removed and the cells were fixed with methanol-acetone (1:1 v/v) for 20 min at -20°C. The NTC, *IGF-I*-TC, *SOX9*-TC, and CTC on the 3rd, 6th, and 9th PT days were incubated with monoclonal antibodies against both Col-I (ab23446; Abcam, Inc., USA) and Col-II (ab34712; Abcam, Inc.), and positive staining was detected using mouse- and rabbit-specific HRP/DAB detection IHC Kit (Abcam, Inc.), according to the manufacturer's instructions.

### Immunolabeling analysis

The immunocytochemistry preparations were examined at 40× with a Nikon microscope (Nikon Instruments) equipped with a digital camera (Labpohot 2; Nikon Instruments) with a resolution of 1600×1200 pixels. Eight fields in each well of the chamber were randomly chosen and imaged. The color photomicrographs were stored in the NIS-elements BR 2.30 software (Nikon Instruments) and digitally binarized. The background was uniformly eliminated with a digital filter and the cell staining intensities were analyzed with ImageJ software (National Institutes of Health, USA). The intensity of the immunolabeling is reported as the means±SD of the total pixels normalized to the number of cells in the measurement area (n=32 in each chamber slide).

### GAG analysis

The accumulated free GAG in the chondrocyte-conditioned culture media from all culture conditions was measured following the dimethyl-methylene blue spectrophotometric assay using the Rheumera GAG detection kit (Astarte Biologics, USA), according to the manufacturer's instructions. Chondroitin sulfate provided with the kit was used as the standard. The total protein concentration was determined by the Bradford method in each assayed culture medium and used to normalize the GAG quantifications.

### Ethics

Written informed consent was obtained from all patients even though this study posed no risk to the patients because it was performed using surplus material donated by patients who were undergoing knee chondrografts. The protocol for this study was approved by the Research and Ethical Committee of the Medicine Faculty of the Universidad Autónoma de Nuevo León (registry number: OR03-053).

### Statistical analysis

All results are reported as mean±SD of three independent experiments performed in triplicate (n=9). Two-way ANOVA with Bonferroni's *post hoc* tests were performed for all experiments except for the stimulation of proliferation experiment, which was assessed using Student's *t*-tests. Statistical analyses were performed in GraphPad Prism version 5.00 for Windows (GraphPad Software, Inc., USA). P-values less than 0.05 were considered to be statistically significant.

## Results

### Transfection efficiency

The efficiency of chondrocyte transfection with pCMV-SPORT6 EGFP was 65.8±10.65 at 3:1, 53.2±4.43 at 3:2, and 52.5±5.13% at 6:1 ratios of FuGENE 6 transfection reagent to pCMV-SPORT6 EGFP. Although no significant differences in transfection efficiency with ratio were found, the 3:1 ratio was chosen for all subsequent transfection experiments.

### Effects of *IGF-I* and *SOX9* on proliferation

On day 3 PT, the NTC, *IGF-I*-TC, *SOX9*-TC, and CTC showed similar numbers of cells per microscope field (19.9±4.2, 20.8±4.4, 20.1±4.9, and 33.0±7.6). However, on day 9 PT, the *IGF-I*-TC cultures contained 47.2 more (153.2±25.6 cells) and the CTC cultures contained 54.8 more (161.1±42.2 cells) cells than the NTC (104.1±19.7 cells) cultures. These increases in the number of cells were both statistically significant (P<0.05).

### Expression of *IGF-I*, *SOX9*, and *GAPDH*

**Figure 1:**
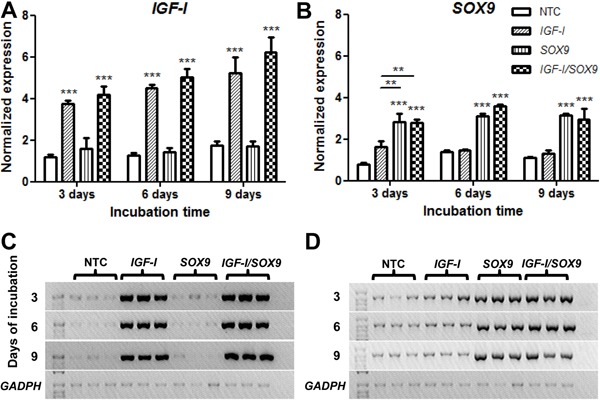
Chondrocyte expression of *IGF-I*, *SOX9*, and *GAPDH*. The panels show *A*, the results of *IGF-I* and *B*, *SOX9* densitometry, and *C* and *D*, cDNA electrophoresis bands. The columns in *A* and *B* indicate the amplicon densitometry from the non-transfected chondrocytes (NTC), or those transfected with pCMV-SPORT6 *IGF-I*(*IGF-I*), pCMV-SPORT6 *SOX9*(*SOX9*), or both plasmids (*IGF-I/SOX9*). Data are reported as the average and standard deviation of the density values normalized to the corresponding *GAPDH* density of 9 assessments. ***P<0.001, NTC *vs IGF-I*, *SOX9* or *IGF-I/SOX9* transfected chondrocytes (two-way ANOVA with Bonferroni *post hoc* tests). Differences in IGF-I/SOX9 and *SOX9 vs IGF-I* on day 3 (**P<0.01) are indicated with lines (two-way ANOVA with Bonferroni *post hoc* tests).

As shown in Figure 1A, the expression of *IGF-I* by the NTC and *SOX9*-TC was not significantly changed from days 3 to 9 PT. In contrast, from the 3rd day until the 9th day PT, significantly higher *IGF-I* expression was found by *IGF-I*-TC (4.2-fold) and CTC (5.5-fold) compared with that of the NTC or *SOX9*-TC. No significant differences in *IGF-I* expression were observed between the *IGF-I*-TC and the CTC or between the *SOX9*-TC and NTC. The expression of *SOX9* was noticeably lower by *SOX9*-TC (2.7-4.1-fold) and CTC (1.5-2.0-fold) compared with their expression of *IGF-I*. The expression of *IGF-I* by CTC on day 9 PT was 3.7-times higher than that by NTC. On days 6 and 9 PT, all cultures maintained a steady level of *SOX9* expression. In addition, throughout the observation period, the expression of *SOX9* was significantly higher by the *SOX9*-TC (3.6-4.2-fold) and CTC (3.5-3.75-fold) than that of the NTC (3.5-3.75-fold), as well as that by the *IGF-I*-TC (2.5-3.7-fold; Figure 1B). The expression of *SOX9* by the CTC on day 9 PT was 2.7 times higher than that by the NTC. The expression of *GAPDH* was not noticeably changed with any culture condition.

### RT-PCR quantitation of *Col-I* and *Col-II*expression

**Figure 2:**
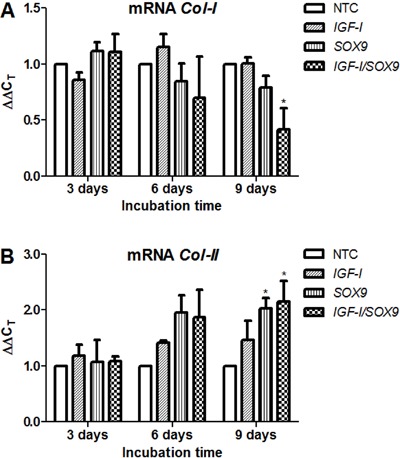
Relative *Col-I* and *Col-II* gene expression by quantitative RT-PCR. Data are reported as the average and standard deviation of the 9 assessments of the expression level of the analyzed genes in non-transfected chondrocytes (NTC) or those that were transfected with pCMV-SPORT6 *IGF-I*(*IGF-I*), pCMV-SPORT6 *SOX9*(*SOX9*), or with both plasmids (*IGF-I/SOX9*). The gene expressions of *Col-I* (*A*) and *Col-II* (*B*) were normalized to the *GAPDH* expression levels. *P<0.05 *vs* NTC (two-way ANOVA with Bonferroni *post hoc* tests).

As shown in Figure 2A, *Col-I* expression appeared to decrease by 16.7% (*SOX9*-TC) and 31.4% (CTC) compared with the NTC expression on day 6 PT, but these differences were not statistically significant. However, the expression of *Col-I* was only significantly decreased in the CTC compared with that expressed by the NTC (59.3%) at day 9 PT. In contrast, the expression of *Col-II*(Figure 2B) showed a clear and consistent increase from days 3 to 9 PT in all cultures. The increased expressions of Col-II by *SOX9*-TC and CTC were more evident on days 6 and 9 PT. However, the cultures only showed significant differences compared with the NTC (*SOX9*-TC: 2.03-fold; CTC: 2.15-fold) on day 9 PT. No significant differences were found between the NTC and the *IGF-I*-TC, *SOX9*-TC, or CTC on day 6 PT (Figure 2B).

### Col-I and Col-II protein detection

**Figure 3:**
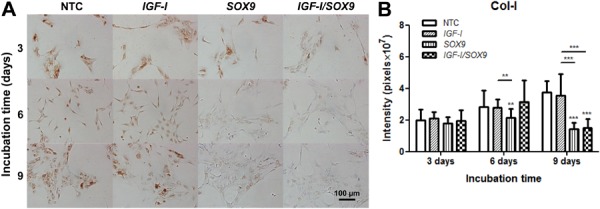
Immunocytochemical detection of Col-I. Specific anti-Col-I primary monoclonal antibodies (*A*) were used to stain the non-transfected human chondrocytes (NTC) and those following transfection with pCMV-SPORT6 *IGF-I*(*IGF-I*), pCMV-SPORT6 *SOX9*(*SOX9*), or cotransfection with both plasmids (*IGF-I/SOX9*). Images were taken on days 3, 6, and 9 post-transfection. Once transfected, the cells were incubated at 37°C in a 5% CO_2_ atmosphere. Quantified *in situ* densitometry is shown in the bar graph (*B*). Data are reported as the average and standard deviation of the densitometry value normalized to the number of cells in the evaluated area (expressed in pixels ×10^7^), which was performed on all chondrocytes observed in 32 images on days 3, 6, and 9 post-transfection. ***P<0.001 and **P<0.01 *vs*NTC; other significant differences are indicated with lines (two-way ANOVA with Bonferroni *post hoc* tests).

**Figure 4:**
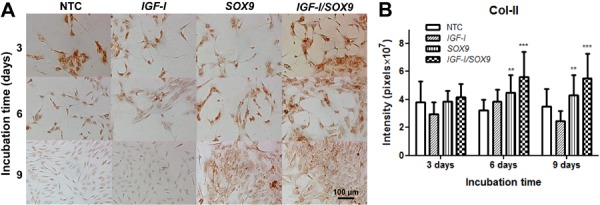
Immunocytochemical detection of Col-II. Specific anti-Col-II primary monoclonal antibodies (*A*) were used to stain the non-transfected human chondrocytes (NTC) and those transfected with plasmids pCMV-SPORT6 *IGF-I* (*IGF-I*), pCMV-SPORT6 *SOX9* (*SOX9*), or following cotransfection with both plasmids (*IGF-I/SOX9*). Images were taken on days 3, 6, and 9 post-transfection. Once transfected, the cells were incubated at 37°C in a 5% CO_2_atmosphere. Quantified *in situ* densitometry is shown in the bar graph (*B*). Data are reported as the average and standard deviation of the densitometry value normalized to the number of cells in the evaluated area (expressed in pixels ×10^7^), which was performed on all chondrocytes observed in 32 images on days 3, 6, and 9 post-transfection. ***P<0.001 and **P<0.01 *vs* NTC (two-way ANOVA with Bonferroni *post hoc* tests).

Immunolabeling for Col-I showed very clear positive staining in all of the chondrocyte cultures on day 3 PT. However, by day 6 PT, the Col-I-positive staining was noticeably diminished in the *SOX9*-TC and CTC cultures and had nearly disappeared on day 9 (Figure 3A). Conversely, all of the cells were intensely positively stained for Col-II throughout the entire observation period (Figure 4A). A morphometric analysis of these image preparations (Figures 3B and 4B) revealed no significant differences among the chondrocyte cultures on day 3 PT. On day 6 PT, the intensity of the Col-I staining was increased in all chondrocyte preparations by 1.2-2.2 times. The greatest increase in the Col-I-positive staining was observed in the NTC, and the lowest increase was noted in the *SOX9*-TC. The staining intensity of Col-I in *SOX9*-TC was significantly lower (25%) than that of NTC. However, no significant differences were found between either *IGF-I*-TC or CTC and NTC. On day 9 PT, the intensities of the Col-I staining had slightly increased in the NTC and *IGF-I*-TC compared with those quantified in those groups on day 6 PT. In contrast, the Col-I staining intensity was significantly decreased in the *SOX9*-TC and CTC (2.7 times in both cases) on day 9 PT. No significant differences were found in Col-I staining between the NTC and *IGF-I*-TC or between the *SOX9*-TC and CTC (Figure 3B).

The morphometric analysis of Col-II (Figure 4B) showed a general increase in the Col-II staining in the *SOX9*-TC and CTC, but not in the NTC. The Col-II staining in the *IGF-I*-TC was lower than in that in the NTC on day 9 PT, but this difference was not statistically significant. On day 6 PT, the Col-II staining was significantly increased in the *SOX9*-TC (1.4 times) and CTC (1.7 times) compared with that in the NTC. The Col-II staining did not increase further on day 9 PT, but remained significantly higher than that in the NTC (*SOX9*-TC: 1.2 times; CTC: 1.6 times). The Col-II staining in the *IGF-I-*TC was lower than that in the NTC, though that difference was not statistically significant.

### GAG production

**Figure 5:**
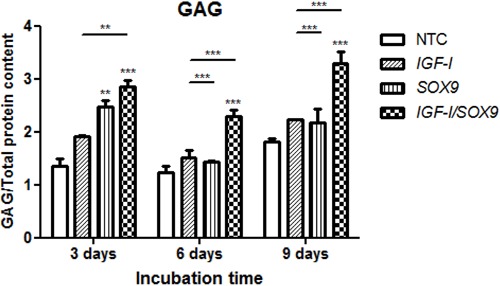
Glycosaminoglycans (GAG) produced by the chondrocytes. The conditioned media from each culture condition were mixed with dimethyl methylene blue dye, and the amount of GAG was quantified by spectrophotometry. The bars correspond to the non-transfected chondrocytes (NTC) and those transfected with pCMV-SPORT6 *IGF-I* (*IGF-I*), pCMV-SPORT6 *SOX9* (*SOX9*), or both plasmids (*IGF-I/SOX9*). Data are reported as the mean and standard deviation of the data normalized to the total protein content based on three experiments on days 3, 6, and 9 post-transfection. ***P<0.001 and **P<0.01 *vs* NTC; other significant differences are indicated with lines (two-way ANOVA with Bonferroni *post hoc* tests).

As shown in Figure 5, all of the chondrocyte cultures (NTC, *IFG-I*-TC, *SOX9*-TC and CTC) accumulated GAG in their respective culture media from days 3 to 9 PT. On day 3 PT, the GAG accumulation was minimal in the NTC culture media compared with that in the *IGF-I*-TC, *SOX9*-TC, and CTC media. The accumulation of GAG in the CTC was consistently higher than those in the other three conditions, and those differences were statistically significant on days 6 and 9 PT (P<0.001). On day 9 PT, the CTC had the largest accumulation of GAG, which was significantly higher than that of the NTC (1.8 times). Significantly more GAG also accumulated in the CTC media than in the *IGF-I*-TC and *SOX9*-TC media.

## Discussion

The results of this study demonstrate that the transitory cotransfection of human chondrocytes with pCMV-SPORT6 *IGF-I* and pCMV-SPORT6 *SOX9* can be achieved with satisfactory efficiency and that cotransfection induced the simultaneous overexpression of both *IGF-I* and *SOX9*. Cotransfection of chondrocytes with these *IGF-I* and *SOX9* vectors resulted in the overexpression of *Col-II* and the reduced expression of *Col-I* compared with *Col-I* and *Col-II* expression by the non-transfected chondrocytes.

We selected 9 days of observation because this period was sufficient to observe the positive expression of the transgenes of interest *in vivo* in several expression systems (18,19). *SOX9* and *IGF-I* were overexpressed in the TC and CTC compared with their respective basal expression by the NTC. This overexpression was maintained throughout the 9-day observation period. The significantly higher number of viable cells in the *IGF-I*-CT and CTC than in the NTC cultures on day 9 is consistent with the well-known concept that *IGF-I* exerts a proliferative effect on growth plate chondrocytes (20). Interestingly, this effect was not found in the presence of cartilage matrix proteins, which is in contrast with the results here for *SOX9-*TC and CTC. This was likely a result of the immature state of the proliferative chondrocytes that were transfected with *IGF-I.*The increasing induction of *Col-II* expression that was observed in the present study occurs physiologically during chondrocyte differentiation (21). The significant reduction in the expression of *Col-I* on day 9 in the CTC suggests a negative transcriptional regulation of *Col-I*. In contrast, it is widely accepted that *SOX9* positively regulates the expression of *Col-II* (22), while *IGF-I* induces *Col-II* transcription and that transcription is mediated by the SOX-trio of *SOX9*, *SOX5*, and *SOX6* (23). These findings strongly suggest that during the proliferation of differentiated chondrocytes that overexpress *SOX9*and *IGF-I*, *Col-II* is preferentially transcribed over *Col-I*. A decrease in Col-I and an increase in Col-II proteins was clearly demonstrated in the CTC over time during incubation. This point is particularly important during articular cartilage repair because the repair tissue that fills the cartilage lesion should possess similar characteristics to hyaline cartilage. *IGF-I* and *SOX9* each individually promoted a small accumulation of GAG in the conditioned media. Together, *IGF-I* and *SOX9* jointly stimulated a significant overproduction of GAG in the CTC compared with that in the NTC. This strongly suggests a synergistic effect of *IGF-I* and *SOX9*. Madry et al. also stimulated Col-II and proteoglycan production (19) by cotransfecting rabbit chondrocytes with *IGF-I* and *FGF-2*, which supports the functionality of two transgenes working simultaneously in the same cells, as we described in the present study.

The transgene expression remained at a higher level than that shown by the NTC during the entire study period. This occurred despite the induction of a transient transfection using plasmids. The transitory effects of *IGF-I* and *SOX9* on transplanted human articular chondrocytes may allow the cells to maintain their phenotype, while also providing anabolic stimuli during cell expansion before transplantation. The apparent synergistic effect of the transgenes together might be explained by the fact that these molecules are expressed together during proliferation and chondrogenic differentiation, which involves the production of proteoglycans and Col-II (24).

To our knowledge, this is the first demonstration that the simultaneous overexpression of *SOX9* and *IGF-I* by human articular chondrocytes induced the overexpression of *Col-II* and the production of GAG, two major components of the human cartilage matrix (25). They also reduced the expression of *Col-I*, a major component of bone, but not of cartilage (17). These advances represent a promising alternative for cell-based therapies of human joint injuries.
